# 2-pyrazoline derivatives in neuropharmacology: Synthesis, ADME prediction, molecular docking and in vivo biological evaluation

**DOI:** 10.17179/excli2017-250

**Published:** 2017-05-08

**Authors:** Savita Upadhyay, Avinash C. Tripathi, Sarvesh Paliwal, Shailendra K. Saraf

**Affiliations:** 1 Division of Pharmaceutical Chemistry, Faculty of Pharmacy, Babu Banarasi Das Northern India Institute of Technology, Lucknow-226028, U.P., India; 2 Professor and Head, Department of Pharmacy, Banasthali Vidyapith, Banasthali, Tonk-304022, Rajasthan, India

**Keywords:** 4,5-Dihydro-(1H)-pyrazoles, antidepressant, anxiolytic, MAO inhibitors, neurotoxicity, microwave synthesis, molecular docking

## Abstract

A novel series of 1,3,5-trisubstituted-2-pyrazoline derivatives **(PFC-1 to PFC-16**) were synthesized in a three step reaction using conventional and microwave assisted green chemistry approach. The synthesized derivatives were characterized and their chemical structures were established by various physicochemical methods such as IR, Mass, ^1^H-NMR, ^13^C-NMR and elemental analysis. The synthesized compounds were tested for their neuropharmacological potential. The compounds exhibited significant antidepressant and anti-anxiety activities against various behavioral *in vivo *models. Compounds **PFC-3** and **PFC-12** were found to be the most active derivatives in the series. The 2-pyrazoline analogs, having 2-hydroxyphenyl and anthracen-9-yl substitution at 3^rd^ position while 4-benzyloxyphenyl and 4-methylphenyl substitution at 5^th^ position, were decisive in eliciting good antidepressant and anxiolytic properties, respectively. The docking experiments revealed that the synthesized derivatives were potential inhibitors of MAO-A protein, which plays a central role in managing depression and anxiety disorders. The most potent derivatives were found to be involved in some key interactions with Tyr407, Tyr444, Phe352 and Ala68 amino acid residues at the binding site of MAO-A protein. All the synthesized derivatives successfully passed the pharmacokinetic barriers of absorption, distribution, metabolism and elimination as predicted using *in silico* techniques without showing any substantial indication of acute and neurotoxicity. This was further confirmed in the laboratory by performing acute toxicity studies as per OECD guidelines.

## Introduction

The monoamine oxidase (EC 1.4.3.2; amine oxygen oxidoreductase) is a FAD-dependent major neurotransmitter degrading enzyme present in the outer mitochondria of neuronal, glial and other cells, that catalyse the aerobic oxidation of structurally diverse xenobiotic arylalkylamine substrates including neurotransmitters and exogenous amines to the corresponding aldehyde and imines with the generation of hydrogen peroxide (Rose et al., 1989[30]). In mammals MAO exists in two isoforms, MAO-A and MAO-B. Serotonin and norepinephrine are preferentially deaminated by MAO-A, which is selectively inhibited by clorgyline. However, MAO-B deaminates phenylethylamine and benzylamine and is selectively inhibited by *l*-deprenil. The proper functioning of synaptic neurotransmission is due to rapid degradation of biogenic amines, which are critically important for the regulation of emotional behaviors and other brain functions (Manna et al., 2002[21]). The undesirable elevation in plasma concentration of MAO substrates and deleterious reactivities shown by MAO-catalyzed reaction products are of major concern in mental health problems.

The early development of MAO inhibitors started with heterocyclic chemotypes containing two or more hydrogen atoms, mostly hydrazine/hydrazido derivatives (Binda et al., 2002[4]). However, they have been withdrawn due to serious side effects such as hypertension crises (cheese reaction), liver toxicity, hemorrhage, blurred vision, dizziness and in some cases death (De Colibus et al., 2005[8]). Pyrazolines are considered as cyclic hydrazine moieties having two adjacent nitrogen atoms within the ring in an envelope conformation. Substituted pyrazolines decorated with different functional groups can be considered as cyclic benzylhydrazine moieties, endowed with MAO inhibitory activity (Edmondson et al., 2004[11]). The stability of pyrazoline ring system and possible substitutions at 1^st^, 3^rd^ and 5^th^ positions by different aromatic and heteroaromatic units inspired the researchers to carry out structural modifications with an array of pharmacological activities such as antimicrobial, anti-mycobacterial, antifungal, anti-amoebic, anti-malarial, anti-inflammatory, anti-depressant, antiepileptic, anticancer, anti-HIV, antitubercular, antibacterial, photoluminescence, polarity probes, insecticidal, antinociceptive, antioxidant, hypotensive, cholesterol inhibitory, cannabinoid (CB1) receptor antagonist, nitric oxide synthase (NOS) inhibitor and MAO inhibitor (Abid and Azam 2005[1]; Abid et al., 2009[2]; Camacho et al., 2004[5]; Havrylyuk et al., 2009[13]; Lange et al., 2005[19]; Patil and Bari, 2014[24]; Prasad et al., 2005[28]; Rathish et al., 2009[29]; Shaharyar et al., 2006[31]; Yar et al., 2009[36]; Zampieri et al., 2008[37]; Zhang et al., 2007[38]; Chimenti et al, 2004[7]; Gokhan-Kelekci et al., 2007[12]).

In order to understand the structural requirements, for both inhibition and selectivity towards the two MAO isoforms (MAO-A/ MAO-B), it was decided to synthesize some novel 1, 3,5 trisubstituted-2-pyrazoline derivatives. The influence on the biological behavior, due to the substitutions of different aromatic/heteroaromatic rings at 1^st^, 3^rd^ and 5^th^ position of the pyrazoline nucleus, was in particular the focus of the study and was further corroborated by the computational studies. In addition to the conventional heating methods, microwave assisted organic synthesis approach was employed to prepare the proposed derivatives. A technique which can be used to rapidly explore “chemical space” and increase the vast interaction of the compounds produced is known as microwave assisted organic synthesis. Finally, to predict the binding affinity and ascertain the interactions of the proposed derivatives with the biological target, molecular docking studies were performed.

## Materials and Methods

The chemicals and reagents for synthesis were procured from S. D. Fine Chemicals and Sigma Aldrich, Mumbai, India and the pre-coated TLC sheets were obtained from Merck Chemicals, India and were used as such. Reagent grade solvents were used and were purified and dried by standard methods. Raga's Scientific Microwave System (Ragatech, Pune, Maharashtra, India) was used for the microwave assisted organic synthesis (MAOS). Melting points were determined by open capillary method and are uncorrected. IR spectra were recorded on Bruker FT-IR, ALPHA-T (Eco-ATR) spectrophotometers, (Bruker Corporation, USA) and the values are expressed in cm^-1^. ^1^H-NMR and ^13^C-NMR spectra were recorded on Bruker Avance-400, FTNMR spectrometer (Bruker, Tech. Pvt. Ltd., USA) at 400 MHz and the chemical shifts are reported in parts per million (δ value), taking TMS (δ 0 ppm for ^1^H NMR) as the internal standard. Mass spectra were recorded on Waters UPLC-TQD Mass Spectrometer instrument (Waters Corporation, USA) using LC-ESI or APCI-MS Technique. Elemental analysis was performed on Perkin Elmer-2400, Series-II Analyzer (Waltham, Massachusetts, USA).

### Chemistry

Synthesis of the proposed 2-pyrazoline analogs was carried out in three steps using conventional methods (stirring and refluxing) as well as an environmentally benign green synthetic approach under the exposure of microwave irradiations. It has been observed that the entire reactions performed using microwave assisted organic synthesis (MAOS) produced compounds in better yields and at a faster rate.

#### General procedure for the synthesis of chalcones (PC1-PC16)

*Conventional synthesis: *Ethanolic KOH (60 %, 10 mL) solution was added with stirring over a period of 15 min-2 hrs to the solution of substituted ketones (0.01 M) and aldehydes (0.01 M). The stirring was continued for 24-48 hrs at a low temperature (0-10 °C). On completion of the reaction, the reaction mixture was poured into ice-cold water, and then neutralized to pH 2 with hydrochloric acid (6N). The yellow colored products obtained were filtered, washed, and re-crystallized from methanol (Jayaprakash et al., 2008[15]; Tripathi et al., 2016[33]).

*MAOS: *Substituted ketones (0.01 M) and aldehydes (0.01 M) were reacted, in presence of hydro-alcoholic solution of KOH (60 %, 10 mL), under microwave irradiation (MWI: 120-280 W, 60-230 s). On completion of the reaction, the reaction mixture was poured into ice-cold water, and then neutralized to pH 2 with hydrochloric acid (6N). The yellow colored products obtained were filtered, washed, and re-crystallized from methanol (Jayashree et al., 2008[16]; Tripathi et al., 2016[33]).

#### General procedure for the synthesis of 3,5-disubstituted-2-pyrazoline derivatives(PS1-PS16)

*Conventional synthesis: *The obtained chalcone derivatives were refluxed for 3-6 hrs with excess of hydrazine hydrate in dry ethanol. The hot reaction mixture was poured in ice-cold water to obtain the crude product, which was washed and re-crystallized from appropriate solvent (ethanol/acetone/ethyl acetate) to afford the respective pyrazoline derivative (Karuppasamy et al., 2010[17]; Tripathi et al., 2016[33]).

*MAOS: *The chalcone derivatives obtained were mixed with hydrazine hydrate (in excess) in dry ethanol under the exposure of microwave irradiation (MWI: 240-350 W; 50-400 s). The reaction mixture was poured in ice-cold water to get the crude product, which was washed and re-crystallized from appropriate solvent (ethanol/acetone/ethyl acetate) to give the respective pyrazoline derivative (Chawla et al., 2010[6]; Insuasty et al., 2011[14]; Tripathi et al., 2016[33]).

#### General procedure for the synthesis of 1,3,5-trisubstituted-2-pyrazoline derivatives (PFC1-PFC16)

*Conventional method: *3,5-Disubstituted-2-pyrazolines (0.001M) were reacted with 0.002 M of 4-chlorobenzenesulfonylchloride by stirring, taking tetra hydro furan (10 mL) as the solvent. Stirring was continued for 1-4 hrs. After completion of reaction, the reaction mixture was poured on a petri plate and solvent was evaporated to dryness. The sticky crude product was re-precipitated using acetonitrile or methanol. Recrystallization was done with acetonitrile or methanol to obtain the pure product. 

*MAOS: *Appropriately substituted 3,5-disubstituted-2-pyrazolines (0.001 M) were reacted with 0.002 M of 4-chlorobenzenesulfonylchloride under of microwave irradiation (MWI: 210-350 W; 80-280 s), taking tetra hydro furan (10 mL) as the solvent. After completion of reaction, the reaction mixture was poured on a petri plate and solvent was evaporated to dryness. The sticky crude product was re-precipitated using acetonitrile or methanol. Recrystallization was done with acetonitrile or methanol to obtain the pure product.

#### Characterization of the synthesized 1,3,5-trisubstituted-2-pyrazoline derivatives

Characterization of the intermediate compounds was done with the help of TLC, melting point and mass spectrometry. However, complete physicochemical (Table 1(Tab. 1)) and spectral characterization was carried out for the final derivatives and the values were in accordance with the proposed derivatives.

*2-[1-(4-Chloro-benzenesulfonyl)-5-(4-hydroxy-phenyl)-4,5-dihydro-1H-pyrazol-3-yl]-phenol* **(PFC-1)**

IR (cm-^1^): N-H str (3531), C-H Ar (3036), C=N str (1596), C-H deform (1491), S=O(1349,1162). ^1^H NMR (DMSO, δ ppm): 2.00-2.06 (dd, *J*_ab_: 15.63 Hz, *J*_ax_: 3.47 Hz, 1H, H_a_), 2.54-2.58 (dd, *J*_ab_: 3.78 Hz, *J*_bx_: 16.24 Hz, 1H, H_b_), 3.70-3.73 (dd, *J*_ax_: 3.51 Hz, *J*_bx_: 16.14 Hz, 1H, H_x_), 5.23-5.27 (s, 2H, Ar-OH), 6.50-7.96 (m, 12H, Ar). ^13^C NMR (DMSO, ppm): 39.2 (CH_2_ pyrazoline), 45.1 (CH pyrazoline), 113.8-146.3 (12CH benzene), 149.6-157.2 (6C benzene), 161.5 (C pyrazoline). MS (*m/z*): 429 (M^+^, 100 %). Anal.Calcd. for C_21_H_17_ClN_2_O_4_S: C, 59.66; H, 4.32; N, 6.32. Found: C, 59.64; H, 4.36; N, 6.29.

*2-[1-(4-Chloro-benzenesulfonyl)-5-(4-methoxy-phenyl)-4,5-dihydro-1H-pyrazol-3-yl]-phenol* **(PFC-2)**

IR (cm-^1^): N-H str (3361), C-H Ar (3049), C=N str (1602), C-H deform (1460), S=O (1349, 1165). ^1^H NMR (DMSO, δ ppm): 2.30-2.48 (dd, *J*_ab_: 17.04 Hz, *J*_ax_: 3.06 Hz, 1H, H_a_), 3.51-3.70 (dd, *J*_ab_: 2.87 Hz, *J*_bx_: 15.24 Hz, 1H, H_b_), 3.22-3.50 (dd, *J*_ax_: 9.14 Hz, *J*_bx_: 15.11 Hz, 1H, H_x_), 7.23-7.86 (m, 12H, Ar), 8.62 (s, 1H, OH). ^13^C NMR (DMSO, ppm): 40.8 (CH_2_ pyrazoline), 47.2 (CH pyrazoline), 76.5 (CH_2_, methylene), 113.4-117.1 (4CH aromatic), 119.1 (2C benzene), 126.8-131.7 (8CH aromatic), 135.2 (C benzene), 144.7 (C benzene), 158.5 (C pyrazoline), 160.4-160.8 (2C benzene). MS (*m/z*): 443.2 (M^+^, 75 %). Anal.Calcd. for C_22_H_19_ClN_2_O_4_S: C, 59.66; H, 4.32; N, 6.32. Found: C, 59.70; H, 4.31; N, 6.35.

*2-[5-(4-Benzyloxy-phenyl)-1-(4-chloro-benzenesulfonyl)-4,5-dihydro-1H-pyrazol-3-yl]-phenol* **(PFC-3)**

IR (cm-^1^): N-H str (3258), C-H Ar (3115), C=N str (1605), C-H deform (1448), S=O (1380, 1162). ^1^H NMR (DMSO, δ ppm): 2.27-2.51 (dd, *J*_ab_: 16.74 Hz, *J*_ax_: 2.99 Hz, 1H, H_a_), 3.67-3.94 (dd, *J*_ab_: 3.02 Hz, *J*_bx_: 14.23 Hz, 1H, H_b_), 3.15-3.63 (dd, *J*_ax_: 9.86 Hz, *J*_bx_: 15.66 Hz, 1H, H_x_), 7.11-7.82 (m, 12H, Ar), 8.62 (s, 1H, OH). ^13^C NMR (DMSO, ppm): 41.5 (CH_2_ pyrazoline), 49.6 (CH pyrazoline), 79.2 (CH_2_, methylene), 112.7-119.8 (4CH aromatic), 118.4 (C benzene), 127.3-130.7 (8CH aromatic), 135.2 (C benzene), 141.6 (C benzene), 157.3 (C pyrazoline), 159.4-160.0 (2C benzene). MS (*m/z*): 519.6 (M^+^, 80 %). Anal. Calcd. for C_28_H_23_ClN_2_O_4_S: C, 64.80; H, 4.47; N, 5.40. Found: C, 64.83; H, 4.45; N, 5.41.

*2-[1-(4-Chloro-benzenesulfonyl)-5-p-tolyl-4,5-dihydro-1H-pyrazol-3-yl]-phenol ***(PFC-4)**

IR (cm-^1^): N-H str (3456), C-H Ar (3083), C=N str (1582), C-H deform (1414), S=O (1365, 1168). ^1^H NMR (DMSO, δ ppm): 2.58-2.71 (dd, *J*_ab_: 17.55 Hz, *J*_ax_: 3.27 Hz, 1H, H_a_), 3.70-3.96 (dd, *J*_ab_: 3.12 Hz, *J*_bx_: 16.23 Hz, 1H, H_b_), 3.11-3.56 (dd, *J*_ax_: 11.47 Hz, *J*_bx_: 16.22 Hz, 1H, H_x_), 4.09 (s, 3H, CH_3_), 7.14-7.91 (m, 12H, Ar), 9.02 (s, 1H, OH). ^13^C NMR (DMSO, ppm): 26.5 (CH_3_, methyl), 41.7 (CH_2_ pyrazoline), 48.6 (CH pyrazoline), 112.7-128.7 (12CH aromatic), 134.2-137.6 (3C benzene), 157.3 (C pyrazoline), 160.1-161.9 (2C benzene). MS (*m/z*): 428.1 (M^+1^, 80 %). Anal. Calcd. for C_22_H_19_ClN_2_O_3_S: C, 61.89; H, 4.49; N, 6.56. Found: C, 61.91; H, 4.53; N, 6.57.

*4-[2-(4-Chloro-benzenesulfonyl)-5-(4-methoxy-phenyl)-3,4-dihydro-2H-pyrazol-3-yl]-phenol* **(PFC-5)**

IR (KBr, cm-^1^): N-H str (3459), C-H Ar (2994), C=N str (1598), C-H deform (1451), S=O (1340, 1213). ^1^H NMR (DMSO, δ ppm): 2.20-2.48 (dd, *J*_ab_: 17.04 Hz, *J*_ax_: 3.29 Hz, 1H, H_a_), 3.56-3.85 (dd, *J*_ab_: 3.17 Hz, *J*_bx_: 15.23 Hz, 1H, H_b_), 3.15-3.63 (dd, *J*_ax_: 9.86 Hz, *J*_bx_: 15.66 Hz, 1H, H_x_), 4.12 (s, 3H, CH_3_), 7.21-7.79 (m, 12H, Ar), 8.70 (s, 1H, OH). ^13^C NMR (DMSO, ppm): 41.5 (CH_2_ pyrazoline), 49.6 (CH pyrazoline), 60.3 (CH_2_, methylene), 115.5-127.3 (12CH aromatic), 128.2-136.4 (3C benzene), 158.6 (C pyrazoline), 159.4-160.0 (2C benzene). MS (*m/z*): 443.7 (M^+1^, 80 %). Anal. Calcd. for C_22_H_19_ClN_2_O_4_S: C, 59.66; H, 4.32; N, 6.32. Found: C, 59.70; H, 4.30; N, 6.33.

*3-Anthracen-9-yl-5-(4-benzyloxy-phenyl)-1-(4-chloro-benzenesulfonyl)-4,5-dihydro-1H-pyrazole* **(PFC-9)**

IR (cm-^1^): N-H str (3301), C-H Ar (3051), C=N str (1608), C-H deform (1452), S=O(1375, 1178). ^1^H NMR (DMSO, δ ppm): 2.60-2.72 (dd, *J*_ab_: 15.98 Hz, *J*_ax_: 3.33 Hz, 1H, H_a_), 3.69-3.87 (dd, *J*_ab_: 3.16 Hz, *J*_bx_: 15.77 Hz, 1H, H_b_), 3.24-3.58 (dd, *J*_ax_: 9.15 Hz, *J*_bx_: 16.86 Hz, 1H, H_x_), 5.11 (s, 2H, CH_2_), 7.43-7.96 (m, 22H, Ar). ^13^C NMR (DMSO, ppm): 41.8 (CH_2_ pyrazoline), 49.6 (CH pyrazoline), 78.3 (CH_2_, methylene), 128.7-134.4 (21CH aromatic), 138.4 (5C aromatic), 160.3 (C pyrazoline), 161.4 (C aromatic). MS (*m/z*):603.1 (M^+1^, 90 %). Anal. Calcd. For C_36_H_27_ClN_2_O_3_S: C, 71.69; H, 4.51; N, 4.64. Found: 71.71; H, 4.47; N, 4.65.

*3-Anthracen-9-yl-1-(4-chloro-benzenesulfonyl)-5-(4-methoxy-phenyl)-4,5-dihydro-1H-pyrazole* **(PFC-10)**

IR (cm-^1^): N-H str (3379), C-H Ar (3105), C=N str (1611), C-H deform (1446), S=O (1359, 1178). ^1^H NMR (DMSO, δ ppm): 3.02-3.11 (dd, *J*_ab_: 17.58 Hz, *J*_ax_: 3.13 Hz, 1H, H_a_), 3.67-3.91 (dd, *J*_ab_: 3.29 Hz, *J*_bx_: 15.44 Hz, 1H, H_b_), 327.1-3.56 (dd, *J*_ax_: 11.45 Hz, *J*_bx_: 16.31 Hz, 1H, H_x_), 3.73 (s, 2H, CH_3_), 7.11-7.82 (m, 17H, Ar). ^13^C NMR (DMSO, ppm): 42.6 (CH_2_ pyrazoline), 47.3 (CH pyrazoline), 57.2 (CH_3_ methyl), 125.7-134.3 (17CH aromatic), 138.4 (7C aromatic), 157.6 (C pyrazoline), 160.0 (C aromatic). MS (*m/z*): 528.5 (M^+1^, 85 %). Anal. Calcd. for C_30_H_23_ClN_2_O_3_S: C, 68.37; H, 4.40; N, 5.32. Found: 68.36; H, 4.43; N, 5.28.

*4-[5-Anthracen-9-yl-2-(4-chloro-benzenesulfonyl)-3,4-dihydro-2H-pyrazol-3-yl]-phenol* **(PFC-11)**

IR (cm-^1^): N-H str (3340), C-H Ar (3050), C=N str (1575), C-H deform (1421), S=O (1380, 1160). ^1^H NMR (DMSO, δ ppm): 2.20-2.35 (dd, *J*_ab_: 16.45 Hz, *J*_ax_: 3.09 Hz, 1H, H_a_), 3.61-3.90 (dd, *J*_ab_: 3.02 Hz, *J*_bx_: 16.23 Hz, 1H, H_b_), 3.15-3.64 (dd, *J*_ax_: 11.06 Hz, *J*_bx_: 15.70 Hz, 1H, H_x_), 7.23-7.91 (m, 16H, Ar), 8.55 (s, 1H, OH). ^13^C NMR (DMSO, ppm): 39.8 (CH_2_ pyrazoline), 49.5 (CH pyrazoline), 116.7-127.3 (17CH aromatic), 135.2 (7C aromatic), 157.3 (C pyrazoline), 162.1 (C aromatic). MS (*m/z*): 528.5 (M^+1^, 65 %). Anal. Calcd. for C_29_H_21_ClN_2_O_3_S: C, 67.90; H, 4.13; N, 5.46. Found: C, 67.92; H, 4.16; N, 5.47.

*3-Anthracen-9-yl-1-(4-chloro-benzenesulfonyl)-5-p-tolyl-4,5-dihydro-1H-pyrazole* **(PFC-12)**

IR (cm-^1^): N-H str (3273), C-H Ar (2925), C=N str (1668), C-H deform (1438), S=O (1360, 1162). ^1^H NMR (CDCl_3_, δ ppm): 2.40 (s, 3H,), 3.23-3.61 (dd, *J*_ab_: 15.28 Hz, *J*_ax_: 4.77 Hz, 1H, H_a_), 5.15-5.22 (dd, *J*_ab_: 6.33 Hz, *J*_bx_: 11.27 Hz, 1H, H_b_), 7.25-7.58 (m, 4H, Ar), 7.77-8.49 (m, 8H, Ar). ^13^C NMR (CDCl_3_, ppm): 21.4 (CH_2_ pyrazoline), 50.7 (C pyrazoline), 76.8-77.6 (2CH aromatic), 124.5-134.3 (14CH aromatic), 136.2-138.8 (6C aromatic), 159.4 (C pyrazoline). MS (*m/z*): 512.3 (M^+1^, 100 %). Anal. Calcd. for C_30_H_23_ClN_2_O_2_S: C, 70.51; H, 4.54; N, 5.48. Found: C, 70.50; H, 4.51; N, 5.44.

*5-(4-Benzyloxy-phenyl)-1-(4-chloro-benzenesulfonyl)-3-(1H-pyrrol-2-yl)-4,5-dihydro-1H-pyrazole* **(PFC-13)**

IR (cm-^1^): N-H str (3195), C-H Ar (3024), C=N str (1602), C-H deform (1444), S=O (1356, 1165). ^1^H NMR (DMSO, δ ppm): 2.26-2.52 (dd, *J*_ab_: 16.73 Hz, *J*_ax_: 2.97 Hz, 1H, H_a_), 3.66-3.90 (dd, *J*_ab_: 3.05 Hz, *J*_bx_: 14.27 Hz, 1H, H_b_), 3.18-3.60 (dd, *J*_ax_: 9.76 Hz, *J*_bx_: 15.61 Hz, 1H, H_x_), 5.47 (s, 1H, NH), 6.14 (s, 3H, pyrrole), 7.12-7.87 (m, 12H, Ar). ^13^C NMR (DMSO, ppm): 39.8 (CH_2_ pyrazoline), 52.5 (CH pyrazoline), 76.2 (CH_2_, methylene), 112.7-129.8 (12CH aromatic), 118.4 (3CH pyrrole), 127.3-130.7 (8CH aromatic), 137.2-139.2 (4C aromatic), 157.8 (C pyrazoline), 159.1 (C aromatic). MS (*m/z*): 492.3 (M^+^, 80 %). Anal. Calcd. for C_26_H_22_ClN_3_O_3_S: C, 63.47; H, 4.51; N, 8.54. Found: C, 63.52; H, 4.49; N, 8.71. 

*1-(4-Chloro-benzenesulfonyl)-5-(4-methoxy-phenyl)-3-(1H-pyrrol-2-yl)-4,5-dihydro-1H-pyrazole* **(PFC-14)**

IR (cm-^1^): N-H str (3382), C-H Ar (3010), C=N str (1600), C-H deform (1440), S=O (1350, 1121). ^1^H NMR (DMSO, δ ppm): 2.18-2.44 (dd, *J*_ab_: 16.22 Hz, *J*_ax_: 3.07 Hz, 1H, H_a_), 3.81-3.93 (dd, *J*_ab_: 3.12 Hz, *J*_bx_: 15.30 Hz, 1H, H_b_), 3.12-3.66 (dd, *J*_ax_: 9.96 Hz, *J*_bx_: 15.21 Hz, 1H, H_x_), 3.51 (s, 3H, CH_3_), 5.28 (s, 1H, NH), 6.39 (s, 3H, pyrrole), 7.17-7.85 (m, 8H, Ar). ^13^C NMR (DMSO, ppm): 39.8 (CH_2_ pyrazoline), 52.5 (CH pyrazoline), 59.8 (CH_3_, methyl), 112.7-129.8 (8CH aromatic), 121.1 (3CH pyrrole), 117.3-126.5 (8CH aromatic), 137.2-138.6 (3C aromatic), 157.8 (C pyrazoline), 160.5 (C aromatic). MS (*m/z*): 416.6 (M^+^, 60 %). Anal. Calcd. for C_20_H_18_ClN_3_O_3_S: C, 57.76; H, 4.36; N, 10.10. Found: C, 57.75; H, 4.39; N, 10.08.

*4-[2-(4-Chloro-benzenesulfonyl)-5-(1H-pyrrol-2-yl)-3,4-dihydro-2H-pyrazol-3-yl]-phenol* **(PFC-15)**

IR (cm-^1^): N-H str (3325), C-H Ar (2997), C=N str (1623), C-H deform (1462), S=O (1214, 1164). ^1^H NMR (DMSO, δ ppm): 2.61-2.84 (dd, *J*_ab_: 15.22 Hz, *J*_ax_: 3.34 Hz, 1H, H_a_), 3.63-3.99 (dd, *J*_ab_: 2.28 Hz, *J*_bx_: 13.91 Hz, 1H, H_b_), 3.44-3.71 (dd, *J*_ax_: 9.58 Hz, *J*_bx_: 14.46 Hz, 1H, H_x_), 5.28 (s, 1H, NH), 6.39 (s, 3H, pyrrole), 7.24-7.68 (m, 8H, Ar), 8.23 (s, 1H, OH). ^13^C NMR (DMSO, ppm): 40.3 (CH_2_ pyrazoline), 49.8 (CH pyrazoline), 114.1-128.5 (8CH aromatic), 116.7 (3CH pyrrole), 135.2-139.6 (3C aromatic), 156.2 (C pyrazoline), 162.1 (C aromatic). MS (*m/z*): 402.8 (M^+^, 75 %). Anal. Calcd. for C_19_H_16_ClN_3_O_3_S: C, 56.79; H, 4.01; N, 10.46. Found:C, 56.82; H, 3.97; N, 10.41. 

*1-(4-Chloro-benzenesulfonyl)-3-(1H-pyrrol-2-yl)-5-p-tolyl-4,5-dihydro-1H-pyrazole* **(PFC-16)**

IR (cm-^1^): N-H str (3378), C-H Ar (3182), C=N str (1605), C-H deform (1438), S=O (1353, 1169). ^1^H NMR (DMSO, δ ppm): 2.08-2.34 (dd, *J*_ab_: 16.02 Hz, *J*_ax_: 3.27 Hz, 1H, H_a_), 3.61-3.95 (dd, *J*_ab_: 3.17 Hz, *J*_bx_: 16.34 Hz, 1H, H_b_), 3.18-3.60 (dd, *J*_ax_: 9.81 Hz, *J*_bx_: 15.29 Hz, 1H, H_x_), 2.51 (s, 3H, CH_3_), 5.33 (s, 1H, NH), 6.06 (s, 3H, pyrrole), 7.13-7.69 (m, 8H, Ar). ^13^C NMR (DMSO, ppm): 39.5 (CH_2_ pyrazoline), 54.6 (CH pyrazoline), 33.7 (CH_3_, methyl), 118.7-126.8 (8CH aromatic), 118.9 (3CH pyrrole), 137.2-138.6 (3C aromatic), 153.8 (C pyrazoline), 155.6 (C aromatic).MS (*m/z*): 401.3 (M^+1^, 90 %). Anal. Calcd. for C_20_H_18_ClN_3_O_2_S: C, 60.07; H, 4.54; N, 10.51. Found: C, 60.11; H, 4.57; N, 10.49.

### Biological evaluation

#### Study animals

Animals (male albino mice of BALB/c strain, body weight ranging between 20-35 mg) were obtained from the animal house of Faculty of Pharmacy, BBDNIIT, Lucknow, U.P., India and kept in polymeric cages under standard living conditions of 26±2 ºC temperature and 55±5 percent humidity, with regular light and dark cycles, having free access to standard food and water. Animals were treated humanely as per the Institutional Animal Ethics Committee (IAEC) guidelines. The pharmacological studies were approved by the IAEC with protocol number BBDNIIT/IAEC/008/2014. 

#### Study design

For the study, animals were divided into 29 groups, each group consisting of 6 animals (n=6). All the mice were treated at respective doses according to body weight. Treatments including standard/vehicle/test compounds were given at 1 mL/100 g body weight per oral (p.o.). Imipramine (10 mg/kg body weight) for antidepressant activity, and Diazepam (2 mg/kg body weight) for anti-anxiety activity was respectively taken as the standard drugs. Carboxy methyl cellulose (0.5 % CMC solution) was taken as the vehicle (control group). The groups and respective doses are given in Table 2(Tab. 2).

#### Antidepressant activity

Forced swim test (FST) and Tail suspension test (TST) in mice model were used to screen the antidepressant potential of the prepared compounds (**PFC1-PFC16**). These behavioral *in vivo *tests have been successfully used to screen and predict the efficacy and potency of various antidepressant treatments including MAO inhibitors (Porsolt, 1981[26]; Willner and Mitchell, 2002[35]).

##### Porsolt's behavioral despair or forced swim test (FST) in mice

Antidepressant potential of the synthesized compounds was tested using Porsolt's behavioral despair test, i.e. FST. Typical immobility behaviors of mice are induced when they are forced to swim in a limited space from where they cannot flee. During the test, mice were plunged into a glass cylinder containing water and it was observed that the animals, after the first 2 min of the initial struggle, became motionless. The duration of stillness was considered when mice started floating in water in a vertical position, with modest activities in order to prevent sinking, and was documented specially for the last 4 min of the 6 min test. This behavior is explained as a state of despair which can be minimized by different therapeutically effective antidepressants, measured by comparing animals treated at numerous doses of test or standard drugs with control (Porsolt et al., 1977[27]; Vogel, 2002[34]). 

##### Tail suspension test (TST) in mice

This procedure is simple, uses objective test situations and the results are congruent with the validated "behavioral despair" test from Porsolt showing sensitivity towards various drug doses. TST is a superficial method and was used to evaluate the antidepressant potential of test compounds, where the mouse is hanged through its tail using a lever. The total 6 min test duration was segregated into agitation and immobility periods. The rodents showed the state of immobility once they were exposed to an inescapable stress. In TST, the potential antidepressants minimize the state of immobility of mice after active and unsuccessful attempts to escape, when compared with the vehicle treated controls. The locomotor stimulant doses of the tested compounds can be differentiated from antidepressant doses, when the period of serenity is studied in conjunction with locomotor activity(Steru et al., 1985[32]; Vogel, 2002[34]). 

#### Anti-anxiety activity

The most frequently used method to study anti-anxiety behavior of the test compounds is the maze model. Elevated plus maze has found a better acceptance in several laboratories in comparison to the other mazes, such as-water maze, Y-maze and radial maze. This test selectively identifies the anxiolytic (open arm exploration time is increased and time spent in closed arms is decreased) and anxiogenic drugs with opposite effects. The values are represented in percent of control. Open arm exploratory time is increased while motor activity is decreased by benzodiazepines and valproates (Vogel, 2002[34]).

##### Elevated plus-maze test

The elevated plus-maze is used to determine the anxiety-related behavior, measured by the extent to which the mouse avoids to visit the open arm of the maze. It is a standard test of fear and anxiety where animals are positioned at the middle of an elevated four-arm maze, in which two arms were open (50 X 10 X 40 cm) and 2 arms are closed (50 X 10 X 40 cm), with an open roof situated on the opposite side. The maze was elevated to a height of 50 cm. After, one hour of oral administration of the standard drug (Diazepam at a dose of 2 mg/kg b.w.), test compound (at doses; 50 and 100 mg/kg b.w.) and control (0.5 % aqueous CMC suspension), the mice were put in the middle of the maze, facing the closed arm. The observations were recorded during the 6 min test duration: time spent in closed arm and total number of the arm entries (Lister, 1987[20]; Pellow et al., 1985[25]).

#### Neurotoxicity study 

Neurotoxicity studies are used to screen the effect(s) of a test substance on the CNS. Several behavioral studies (such as rotarod test, open-field/actophotometer test, turning on flat surface and turning on inclined plane) are employed to identify potential neurotoxicity in mice model. Rotarod and actophotometer tests are the two most often used methods to identify the effect of chemical compounds (test compound) on neuromuscular coordination and locomotor activities, respectively (Parasuraman, 2011[23]).

##### Neuromuscular coordination study (rotarod test)

The rotarod test is valuable to find out the consequences of drug on motor coordination, i.e. neurotoxicity. This test was introduced by Dunham and Miya in 1956[10], where relaxation of skeletal muscle produced by any test compound is examined by maintaining the ability of mice or rats on a rotating rod, at a speed of 10 revolutions per min. For the test, male mice of average weight 25±5g underwent a pretest, and only those mice were selected for the test which remained on the revolving rod for at least 1 minute. The mice were placed on the rod after 1 hour of oral administration of test compounds/standard drug/control. At this speed, normal mice stay on the rod for an indefinite period of time. However, if they fall before 1 min, the sign of neurotoxicity is apparent at the tested doses. Therapeutic end point is considered as a dose which persuades ability of fifty per cent of the mice to stay on the revolving rod. Number of animals falling down from the rod is counted in a qualitative manner and mean fall off time is recorded to quantify the values (Bhandari et al., 2013[3]; Vogel, 2002[34]).

##### Locomotor activity (actophotometer/open field test)

Locomotor activities of animals, and humans, are affected by majority of the CNS acting drugs and therefore it is utilized as an index of their mental alertness. Some CNS depressant drugs like barbiturates and alcohols have been found to reduce it. However, CNS stimulants like amphetamines and caffeine exhibit significant increase in the motor activity. The locomotor activity was examined for a 10 minute duration, using Medicraft Actophotometer (Model No. 600-4D, INCO, Ambala, India), to rule out such CNS depressant or stimulant type of effects of the tested compounds. During the test, animal moves in a square arena of dimension 30x30x25 cm with mesh wire bottom. Photoelectric cells connected in circuit with a counter are used to operate the apparatus, where light beam striking on the photo cell is cut off by the animal to record a count (Dhingra and Goyal, 2008[9]).

#### Acute toxicity study

Acute toxicity testing is carried out to determine the effect of a single dose on a particular animal species. In the present study, acute oral toxicity (LD_50_) of two most active final derivatives (**PFC-3** and **PFC-12**) was performed as per guidelines laid by Organization for Economic Co-operation and Development (OECD) guideline No 423 “Acute Oral Toxicity - Acute Toxic Class Method”. The compounds were administered orally at four fixed dose levels (5, 50, 300 and 2000 mg/kg body weights), after 4 hours of fasting. After dose administration, food but not water was withheld for 2 hours. The body weight of all the mice were recorded on study days D_0_ (initiation), D_1_, D_7_ and D_14_. The animals were observed for 4 hours post dose treatment and thereafter for 14 days for mortality, including various signs of toxicity such as, changes in skin and fur, behavior patterns, convulsions, tremors and death (OECD, 2001[22]; Parasuraman, 2011[23]).

### Molecular docking studies

Molecular docking simulations were used to predict binding affinity and binding orientations of the synthesized compounds with MAO-A protein (PDB ID: 2Z5X), using GLIDE program. The X-ray crystal structure of MAO protein was retrieved from the Protein Data Bank (PDB) and optimized using “protein preparation wizard”. Ligands were prepared by LigPrep module 3.2 version v25111(Schrödinger, LLC, New York, NY, 2014), using OPLS (optimized potential liquid simulations) 2005 force field to give the consequent energy minima. Default settings were applied to other parameters. Rigid docking was performed during these calculations. Re-docking experiment was conducted to validate the docking procedure, in which the coordinates of crystal ligand were obtained from the PDB structure of MAO-A protein-ligand complex and docked back into the binding pocket. GLIDE (version 6.5, Schrödinger, LLC, New York, NY, 2014) program perfectly reproduced the tentative pose of the ligand and accurately predicted the binding mode.

#### In silico prediction of pharmacokinetic properties

Majority of the drug candidates do not succeed in clinical trials due to poor pharmacokinetic properties, such as absorption, distribution, metabolism, and excretion (ADME). The outlay in developing new drug significantly increases due to these later-stage failures, which can be radically reduced if the problematic candidates are detected in early phases. In this study, the QikProp module (version 4.2) of Schrödinger (LLC, New York, NY, 2014) software program was successfully employed for *in silico* prediction of ADME properties of the synthesized derivatives.

#### In silico toxicity prediction 

There are a large number of freely available computer programs to envisage the *in silico *toxicity of the compounds. Two of these softwares, used in this study are; LAZAR and OSIRIS Property explorer. LAZAR provides a generic tool for predicting complex toxicological end points, such as carcinogenicity, long-term toxicity, and reproductive toxicity. Virtual Computational Chemistry Laboratory maintains OSIRIS as a fundamental part of Actelion's in house substance registration system. This calculates various drug-related properties of chemical structures including some toxicity parameters and drug likeness. Predicted outcomes are rated and color coded. Properties such as mutagenicity or a poor intestinal absorption having higher risks of undesired effects are shown in red, whereas a green color indicates drug-conform behavior (Klebe, 2000[18]).

### Statistical analysis

All the values of the experimental results are expressed as mean±SD and analyzed by one-way ANOVA, followed by Dunnett's test for the possible significance (P<0.05) identification between various groups. Statistical analysis was carried out using Graph Pad Prism 5.0 (Graph Pad Software, San Diego, CA). 

## Results and Discussion

**Figure 1:**
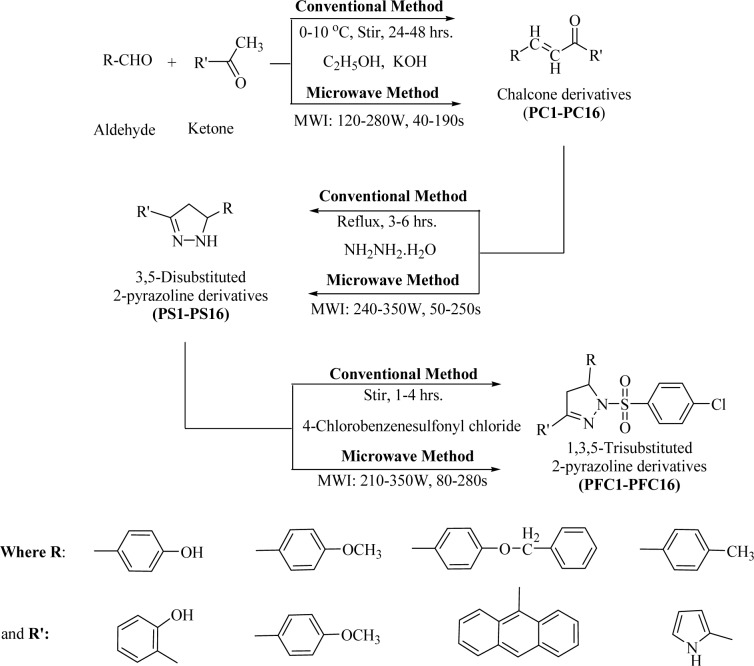
Synthesis of 1,3,5-Trisubstituted-2-pyrazoline derivatives

A series of thirteen 2-pyrazoline derivatives **(PFC1 **to **PFC-16) **were synthesized using conventional as well as a green chemistry approach of microwave assisted organic synthesis (MAOS) as given in Figure 1(Fig. 1). Among the two methods, MAOS showed better synthetic efficiency when compared with the conventional procedures. The synthesized derivatives were characterized by various physicochemical (Table 1(Tab. 1)), spectral and elemental analysis methods. The spectral analysis data expound C=N stretching (1509-1612 cm^-1^), N-H stretching (3456-3105 cm^-1^) and C-H deformation (1428-1357 cm^-1^) along with a characteristic peak of sulfonyl group absorption bands (1340-1180 cm^-1^) in the corresponding regions of IR spectra.

The presence of two non-equivalent protons of a methylene group (H_a_/H_b_) at δ 2.92-3.38 ppm and 3.70-3.93, coupled with each other and in turn with the vicinal methine proton (H_x_) at δ 6.68-7.04 were recorded in the ^1^H-NMR spectra. All the other protons of various aliphatic/aromatic/heteroaromatic groups were anticipated at respective places.

**Figure 2:**
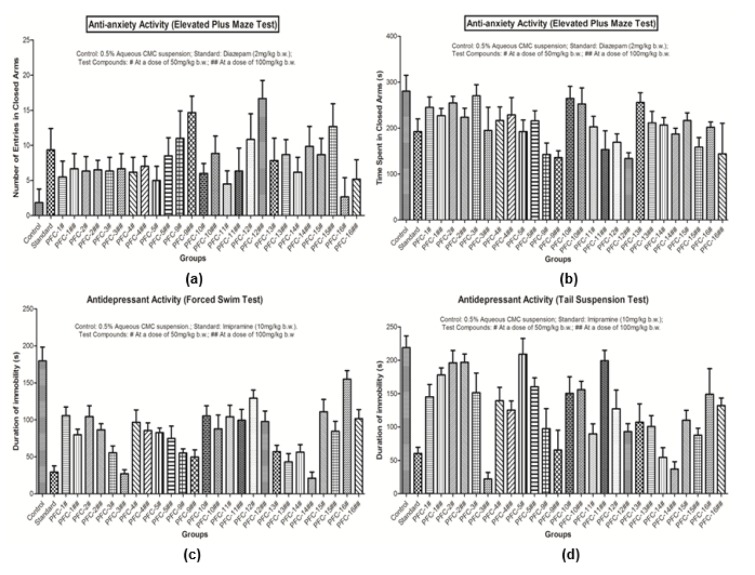
(a) Graph showing anti-anxiety activity (Number of entries in closed arms in Elevated Plus Maze Test); (b) Graph showing anxiety activity (Time spent in closed arms in Elevated Plus Maze Test); (c) Graph showing antidepressant activity (Forced Swim Test); (d) Graph showing antidepressant activity (Tail Suspension Test).For each group, number of animals tested was 6 (n=6).

Results of the pharmacological studies demonstrated that the synthesized 1,3,5-trisubstituted-2-pyrazoline derivatives possess good range of antidepressant and anti-anxiety activities, as evaluated using various *in vivo *models. Compounds **PFC-3 **and **PFC-12 **were found to be the most active compounds amongst all the synthesized derivatives (Table 2(Tab. 2) and Figure 2(Fig. 2)). 

Detailed SAR studies revealed that 4-chlorobenzenesulfonyl substitution at N1 position was decisive in exhibiting good antidepressant and anti-anxiety activities. It was also observed that 2-hydroxyphenyl substitution at 3^rd^ position and 4-benzyloxyphenyl substitution at 5^th^ position of 2-pyrazoline nucleus was instrumental for the remarkable antidepressant potency of compound **PFC-3. **However, a bulky hydrophobic anthracen-9-yl substitution at 3^rd^ position and 4-methylphenyl substitution at 5^th^ position of the 2-pyrazoline scaffold were favorable in demonstrating the significant anxiolytic profile of compound **PFC-12**. The biological activities of the synthesized compounds were depending upon the doses administered, with increased effects at higher tested dose (100 mg/kg b.w.).

All the synthesized derivatives were also evaluated for their possible neurotoxicological effects such as neuromuscular coordination (using rotarod test) and locomotor activity (using actophotometer test). It was observed that none of the synthesized compounds showed serious neurotoxicity (disturbances in the motor co-ordinations and locomotor activity) threats at the tested doses (Table 2(Tab. 2)). Therefore, any possibility of CNS stimulating or depressing effects of the tested compounds was ruled out. Additionally, two most potent derivatives (**PFC-3 **and **PFC-12**) were screened for their acute toxicities, at the tested doses. During these studies; behavioral pattern, changes in skin and fur, convulsions, tremors and death were not observed in any of the animals, in due course of 14 days of acute toxicity studies, as per the OECD guidelines.

**Figure 3:**
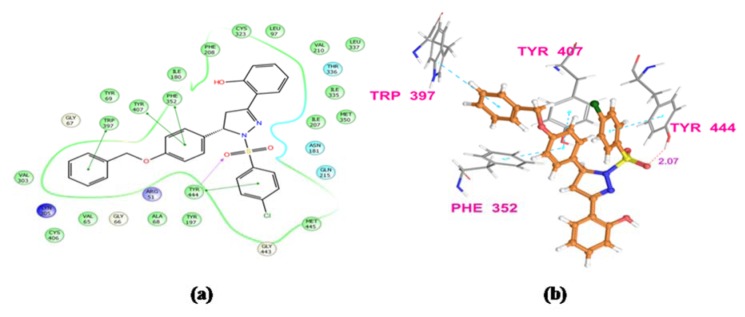
Ligand receptor interaction diagram of compound PFC-3 at the binding site of MAO-A protein (PDB ID: 2Z5X) showing best anti-anxiety activity. (a) 2D Ligand receptor interaction diagram, (b) 3D Ligand receptor interaction diagram

**Figure 4:**
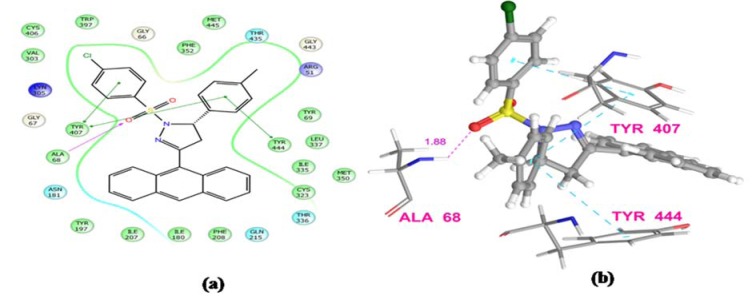
Ligand receptor interaction diagram of compound PFC-12 at the binding site of MAO-A protein (PDB ID: 2Z5X) showing best antidepressant activity. (a) 2D Ligand receptor interaction diagram, (b) 3D Ligand receptor interaction diagram

The results obtained by animal experimentation were further supported by the outcomes of molecular docking experiments. The antidepressant and anxiolytic properties of substituted 2-pyrazoline derivatives may be attributed to their MAO-A inhibiting capabilities. The synthesized compounds were found to possess tremendous affinity towards MAO-A enzyme, and may therefore be deemed as imperative targets to develop potential neuropharmacological agents. Docking studies also confirmed that the presence of sulfonyl group at N1 position of pyrazoline nucleus establishes a hydrogen bonding interaction between the sulfonyl oxygen of the ligands and hydroxyl hydrogen of either Tyr444 or Tyr407 amino acid residue. Due to these Hydrogen bonding interactions, substituted phenyl ring at C5 and benzenesulfonyl ring at N1 position of pyrazoline nucleus are well placed in the aromatic cage. The most potent compound from N1 benzenesulfonyl substituted series (**PFC-12**) displayed additional key interactions of this moiety with side chain and backbone residues of the amino acids at the binding pocket. This included a hydrophobic interaction of benzene ring with Tyr 407, Tyr444 residues, and H-bond interaction with Ala68 and Tyr444 residues (Figure 3(Fig. 3) and 4(Fig. 4)).

It was observed that one amongst the other aromatic substitutions of previously mentioned compounds at these positions is involved in hydrophobic and probably aromatic interaction. Also, favorable *in silico* ADME performances (Table 3(Tab. 3)) were obtained for all the synthesized compounds. Furthermore, the tested compounds were found to be safe, by not presenting any potential risks of carcinogenicity, mutagenicity, reproductive toxicity, acute toxicity and irritancy as predicted by LAZAR and OSIRIS Property Explorer programs (Table 4(Tab. 4)).

## Conclusion

In the present study, *in vivo *and *in silico *screening results have clearly demonstrated the expediency of synthesized 2-pyrazoline derivatives in neuropharmacological disorders. Although, the evidence showed promising status of the tested compounds against *in vivo *models of depression and anxiety, further experiments including *in vitro *MAO-A inhibition studies are needed to explore their concrete mechanism of action at molecular level. Since, all these derivatives were found to be effective in a dose dependent manner, further therapeutic dose adjustment studies and designing of most appropriate dosage forms are some imperative prospects. Some of the excellent outcomes obtained from the preclinical investigations demand comprehensive clinical trials to establish the role of synthesized 1,3,5-trisubstituted-2-pyrazoline derivatives in the management of depression and anxiety disorders. 

## Acknowledgements

We express our sincere gratitude to Central Drugs Research Institute (CDRI), Lucknow, India and Department of Chemistry, Banasthali Vidyapith University, Banasthali, Rajasthan, India for providing the library and sophisticated analytical instrument facilities. Authors are thankful to the All India Council for Technical Education (AICTE), New Delhi, India, for providing grant under the Research Promotion Scheme (Grant No.: 8023/RID/RPS/30 (Pvt.) 2011-12), through which the computational software facility has been made available at the host institute. We also acknowledge the technical support team/application scientists of Schrodinger Inc. for their help during computational studies.

## Conflict of interest

The authors declare that they have no conflict of interest.

## Figures and Tables

**Table 1:**
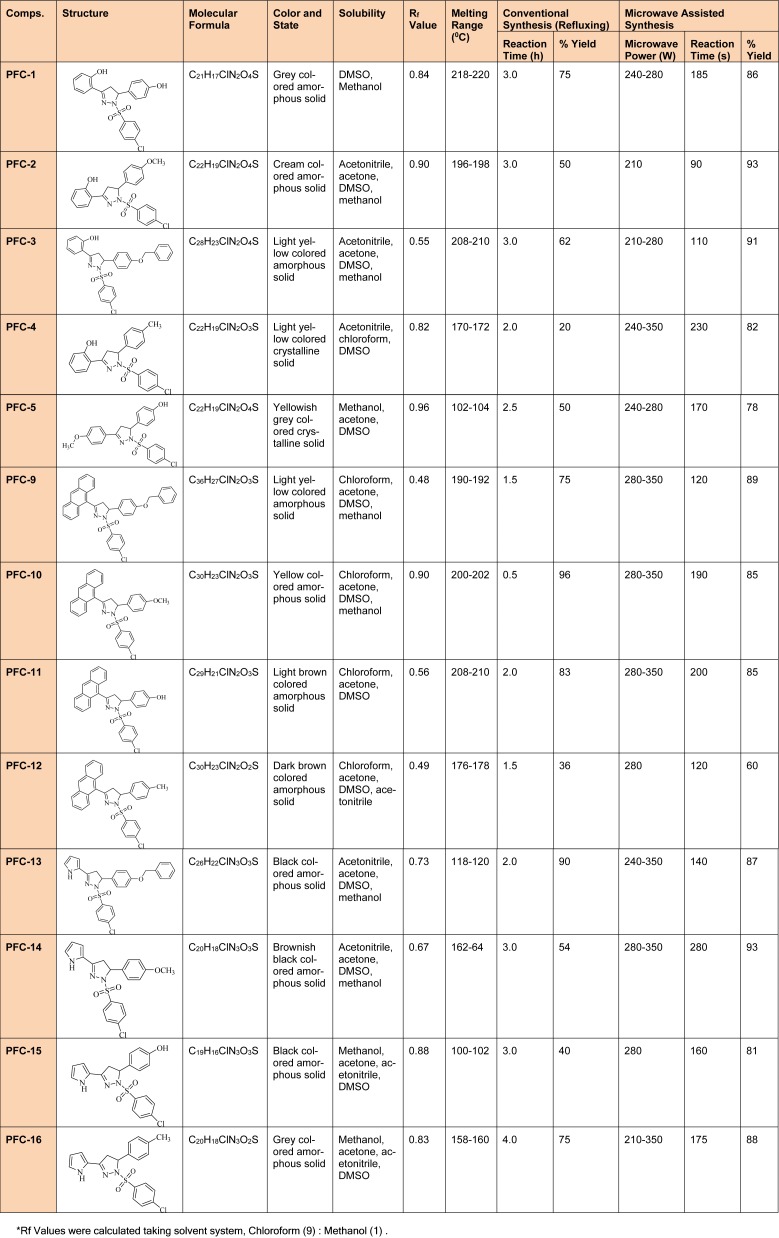
Comparative study of physicochemical properties of synthesized 1,3,5-trisubstituted -2-pyrazoline derivatives (PFC-1 to PFC-16) using conventional and microwave methods

**Table 2:**
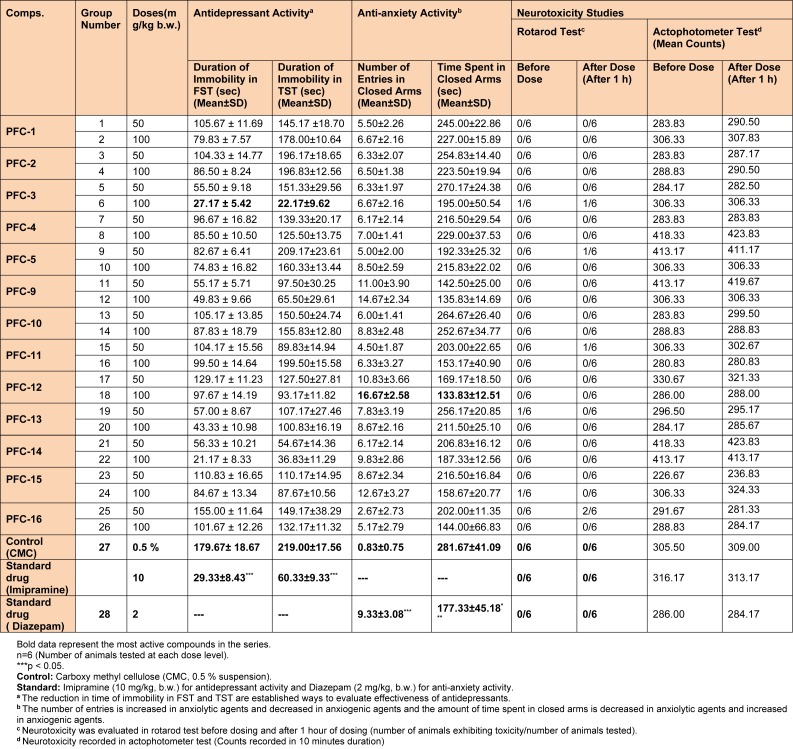
Data showing antidepressant, anti-anxiety and neurotoxicity studies of the synthesized 1,3,5-trisubstituted-2-pyrazoline derivatives (PFC-1 to PFC-16).

**Table 3:**
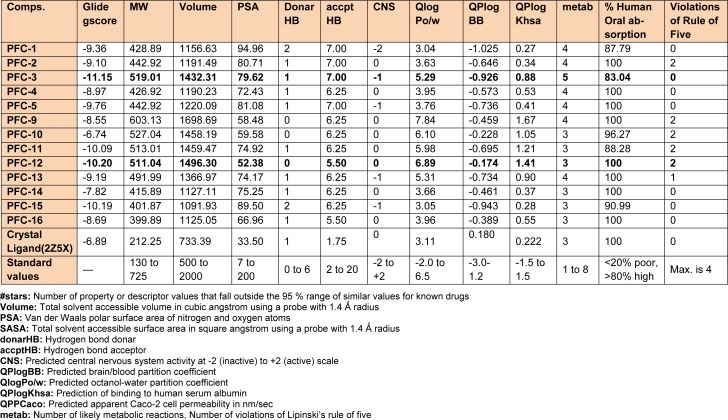
*In silico* prediction of binding affinity (Glide gscore) and ADME parameters of the synthesized 1,3,5-trisubstituted -2-pyrazoline derivatives (PFC-1 to PFC-16)

**Table 4:**
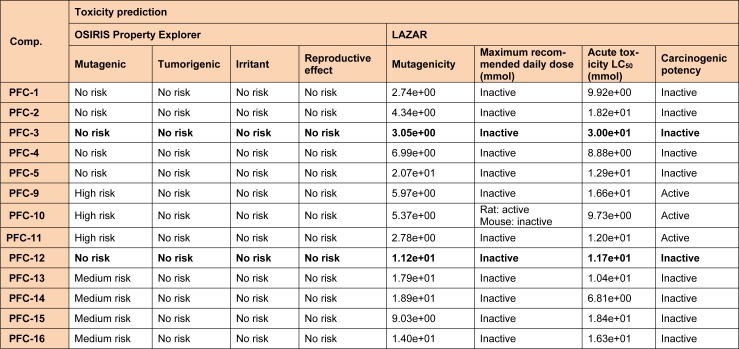
*In silico* toxicity prediction data of the synthesized 1,3,5-trisubstituted -2-pyrazoline derivatives(PFC-1 to PFC-16)
